# Effects of whole-body ELECTROMYOSTIMULATION on health and performance: a systematic review

**DOI:** 10.1186/s12906-019-2485-9

**Published:** 2019-04-24

**Authors:** Alvaro Pano-Rodriguez, Jose Vicente Beltran-Garrido, Vicenç Hernández-González, Joaquim Reverter-Masia

**Affiliations:** 1 Research Group Human Movement, University of Lleida, Av. de l’Estudi Generaln.4 E-25001Lleida, Lleida, Spain; 2 EUSES Health and Sport Sciences School, Rovira i Virgili University, Tarragona, Spain

**Keywords:** Global-body electromyostimulation, Whole-body electrical muscle stimulation, Whole-body electrostimulation, Integral electrical stimulation

## Abstract

**Background:**

Whole-body electrical myostimulation (WB-EMS) is a relatively recent training methodology that has been extraordinarily used in recent years. However, there is a lack of consensus on the effectiveness of WB-EMS in the situations in which its use has been largely popularized. The objective of this systematic review was to determine the effects produced by WB-EMS.

**Methods:**

A search of PubMed, Web of Science, Scopus and Cochrane was performed to identify all the studies that have applied electrical stimulation in lower and upper limbs simultaneously and that have clearly presented their protocols for the training and application of the stimulation. The last search was performed on September 9, 2018. Studies written in English or German were included.

**Results:**

A total of 21 articles met the inclusion criteria and were analyzed following the guidelines of the Cochrane Guide for Systematic Reviews. Nineteen studies analyzed the chronic effects of WB-EMS, and 2 analyzed acute effects with a total of 505 subjects (310 men and 195 women). In total, 35% were moderately trained, and 65% were sedentary subjects. Different dependent variables were studied, such as anthropometric parameters, strength parameters, energy expenditure, psychophysiological parameters and blood parameters. There is a lack of randomized controlled studies, and the studies included exhibit a moderate to high level of risk of bias.

**Conclusions:**

Given the limited number of available studies on WB-EMS, the scarce amount of scientific evidence found does not allow definitive conclusions about its effects; therefore, future studies about WB-EMS are necessary.

**Electronic supplementary material:**

The online version of this article (10.1186/s12906-019-2485-9) contains supplementary material, which is available to authorized users.

## Background

Whole-body electrical myostimulation (WB-EMS) is a relatively recent training methodology that has been extraordinarily lavished in recent years. WB-EMS, which is also called global-body electrical myostimulation, has emerged as the evolution of traditional electrical muscle stimulation (EMS) applied locally, since it is now possible to activate several muscle groups in a synchronized manner as a result of technological development. Using a wireless electrical stimulator that has a powerful battery, it is possible to activate up to twelve channels with a rectangular, two-phase and symmetrical current [1]. These channels generally allow the activation of the muscles of the thighs, arms, buttocks, abdomen, chest, and low, high and lateral areas of the back with two auxiliary channels of free choice and a total area of electrodes up to 2800 cm^2^ [2]. These devices are managed by software that allows the modification of the current parameters and the intensity of each of the channels.

Local EMS is based on the application of the current to the motor point of one or two muscle groups, whereas the WB-EMS procedure is based on doing the same across a large area and along several muscle groups. On the one hand, the application of the current to a motor point during the EMS means that less energy is required to cause the involuntary contraction; therefore, the method is more comfortable [3]. On the other hand, the application of current in a large number of muscle groups in a synchronized manner in WB-EMS makes it possible to exercise complete kinetic chains in unison and perform exercises with global positions and movements during the electrical stimulus [4]. In the WB-EMS, the coactivation of agonist-antagonist muscles is generally observed. This feature may be an advantage given that stimulating an antagonist muscle can contribute to the improvement of aerobic strength and capacity without presenting damage to the motor pattern as shown in previous experimental studies [5, 6].

To date, vast and extensive research has been performed in the study of the effects of local EMS [7–9] that should be taken into account for WB-EMS exercise. It would not be surprising if, despite these slight differences in the two methodologies, future research demonstrates that WB-EMS offers results similar to those obtained with the local EMS for the rehabilitation of injuries [10, 11], i.e., for the effective treatment of spasticity in subjects with neurological disorders [12], exercise for individuals with illnesses [13–15], and for strength training in healthy subjects [4].

It has been concluded that WB-EMS could be an interesting training methodology for people who experience difficulties when exercising given the amount of effort necessary to create adaptations [16]. WB-EMS has also been considered as an alternative with great efficiency in terms of the time-benefit ratio with a high acceptance rate even in untrained individuals [17]. However, other studies have obtained less promising results, presenting a less optimistic position regarding the effectiveness of this type of training [18].

EMS is capable of generating muscle tension greater than that which can occur in voluntary contraction and therefore can cause muscle degradation far superior to what traditional exercise is capable of causing [19]. Therefore, it has been indicated that the use of WB-EMS could be a danger mainly for untrained people, arguing that increasing the number of affected muscle groups could be a risk factor. In fact, over recent years, different case reports have appeared in which rhabdomyolysis has occurred after a training session with an alarming increase in creatine kinase (CK) activity [20–22].

There is a lack of consensus on the effectiveness of WB-EMS in a situation in which its use has been popularized to a large extent, thus increasing the need for a systematic review with the purpose of analyzing the results obtained from the existing research on WB-EMS and testing the level of evidence of each of the studies to understand the status of the issue and identify possible methods of investigation in the future.

## Methods

This review was made from the analysis of the most relevant studies on the subject from an objective and critical perspective. This study was designed following the indications provided by the Cochrane Handbook for Systematic Reviews of Interventions [23] and the Preferred Reporting Items for Systematic Reviews and Meta-Analysis (PRISMA) [24].

### Search strategy and data sources

The first search was conducted on May 15, 2017, whereas the last search was on September 9, 2018. The following databases were used: PubMed/MEDLINE, Scopus, Cochrane and Web of Science. Following the guidelines of each of the databases, the following search strategy was used: {EMS OR whole-body electromyostimulation OR global body electrical stimulation} AND {Fitness OR Hormonal OR Power OR Bone mineral density OR Body composition OR Endurance OR Strength OR Obesity}. The words “EMS”; “Whole-Body Electromyostimulation” and “global body electrical stimulation” were used to identify appropriate (MESH) terms, but any of the results that were consistent with the aim of the study were assessed. In addition, a manual search was performed using the bibliographic lists of the included articles to identify additional studies.

### Inclusion/exclusion criteria

Only articles published in peer-reviewed journals of the mentioned databases without limitations on the publication date were included in this review. Studies were analyzed without restrictions regarding the time of follow-up or intervention. Only studies that analyzed human subjects without limiting their sex, age or physical condition were taken into account. The participants of these studies could have a good health status or suffer from a disease for which WB-EMS was applied as a possible treatment; afterwards, an analysis of the effect of its application on the symptoms of this disease was performed. In addition, the studies included in this review should apply whole-body electrical stimulation in the lower and upper limbs simultaneously as an intervention in at least one group of the sample population. Randomized and nonrandomized clinical trials with control or other equivalent comparison group (control group (CG) or comparison groups formed by subjects who had a different treatment or groups that did not perform any type of physical activity) were included (Table 1).

**Table 1:** PICO criteria details of the systematic review

P	I	C	O
Human subjects without limiting their sex, age or physical condition	EMS Whole-body electrical stimulation Global body electrostimulation applied in the lower and upper limbs simultaneously	Control group Comparison group	Fitness Hormonal Power Mineral density Body composition Endurance Strength Obesity

Exclusion criteria were applied to publications in which the complete article was not included or was any of the following types of articles: letters to the editor, book chapters, unpublished reports, case studies and descriptive retrospective reports.

### Data extraction

The studies in this review analyzed the variables that are reflected in Table 2.

**Table 2:** Variables analyzed in the studies included in the systematic review

Body composition (X-rays or DEXA skinfolds)	Energy consumption and cardiovascular system	Evolution of hormonal and blood parameters	Musculoskeletal and motor system	Indices for the assessment of diseases	Psychophysiological parameters
Bone mineral density (BMD) in the lumbar spine or proximal process of the femur	Maximum oxygen consumption (VO2max)	Testosterone	Isometric Maximal strength (IMS) of manual grip and trunk and leg extenders	Sarcopenia (Sarcopenia Z-score)	Soreness
Body weight	Oxygen consumption after exercise (VO2)	Growth hormone (GH)	Dynamic strength of leg extenders	Metabolic Syndrome (Metabolic Syndrome Z-score)	Anxiety
Body fat	Deformability of red blood cells	Creatine kinase	Running speed in 10 m		Fatigability
Abdominal fat	Basal metabolism at rest	Lactic acid	Running Economy		Sleeplessness
Fat leg	Blood pressure	Cortisol	Countermovement Jump		
Total muscle mass		Triglycerides	Abalakov Jump		
Appendicular musculature		Hemoglobin saturation	Squat Jump		
Fat mass		Total cholesterol/HDL-C ratio			
Body mass index					
Circumferences of hip and waist

**Table 3:** Characteristics of the studies included in the review with a questionable control group

Author and year	Objective and type of study	N	Sample Characteristics	Duration	Weekly sessions	Current parameters	Current intensity	Training protocol
Wolfgang Kemmler 2015	Determine the increases in CK concentration and its corresponding impact on health parameters and changes in concentration levels throughout training	*N* = 11	Men trained but without experience in WB-EMS	10 weeks	1 sessions per week	Bipolar 85 Hz duty cycle: 60% 6–4 s On time of pulse 350 μs Impulse rise: 0 sImpulse decay: 0 s	Intensity ≥7 (very hard) RPE-10 Every 3 min increases by 2–3%	
	Increase of CK after a first WB-EMS session compared with a marathon race	N = 26	Men trained but without experience in WB-EMS Marathoners training level 3 days per week for at least 12 months	Acute effect during 5 days contiguous to the effort				
Wolfgang Kemmler 2012	Analyze the energy expenditure added by the use of WB-EMS	N = 19	Active men students 5 to 8 h of exercise a week last 2 years	One session (16 min)	–	Bipolar 85 Hz duty cycle: 50% 4–4 s On time of pulse 350 μs Impulse rise: 0 sImpulse decay: 0 s	Maximum tolerance	Same exercises from Test I of 2010
Miguel Ángel De la Cámara 2018	Evaluation of WB-EMS as a post-exercise recovery method	N = 9	Trained men 21 years	One session 20 min	–	1 Hz duty cycle: 100% On time of pulse 350 μs Impulse rise: No dataImpulse decay: No data	The most comfortable possible	Subjets lay quietly in a supine position

*WB-EMS (whole-body electrical myostimulation); N (sample size); RPE (rated perceived exertion); R (recovery between series)*

**Fig. 1:**
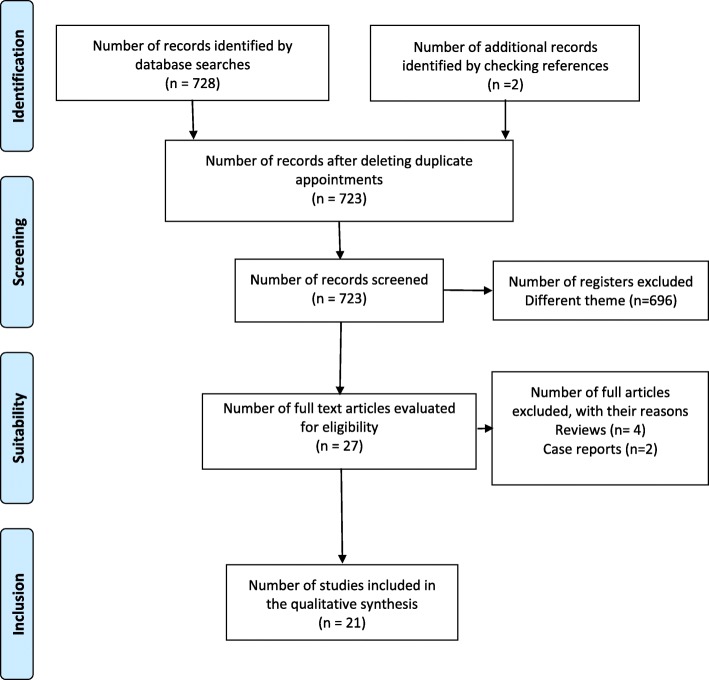
PRISMA flow chart. ﻿Abbreviation: PRISMA, Preferred Reporting Items for Systematic Reviews and Meta-Analysis

The results of the search were imported into the bibliographic management software (Mendeley Desktop® version 1.17.9 for Windows), and duplicates were removed. Then, a rapid assessment was performed to analyze and discard the articles based on titles or abstracts that clearly led to their exclusion. Subsequently, the articles with potential were completely read to determine if they were suitable for inclusion in the review. The selection process that was applied to the articles that were studied was based on the selection criteria mentioned above, including types of intervention, types of variable measurement and types of protocol. The results of the entire search, screening and selection process are presented in the PRISMA diagram (Fig. 1).

All data were extracted from the articles and analyzed using the Cochrane manual extraction tool for systematic reviews of interventions [23]. The following relevant aspects were included:

- Title
- Purpose of the study
- Authors
- Magazine
- Year of publication
- Size and characteristics of the sample
- Groups that comprised the sample
- Intervention received by each group (training program)
- Duration of the intervention
- Training sessions per week
- Parameters of the current
- Results of each of the measurements that were made in the study that could be relevant for this review.

### Risk of bias assessment

A complete assessment of the level of risk of bias of the included studies was made following the guidelines of Higgins & Green [23]: low risk, high risk or unclear risk. For this assessment, it was observed whether the following measures were performed in the preparation of the studies to avoid the different types of bias: randomization of the sample (selection bias), blinding of the sample (performance bias), blinding of the assessors (detection bias), complete reporting of results (attrition bias), and selective reporting of results (notification bias).

Two reviewers (ADP and VBG) conducted the article searches, data extraction and assessment of risk of bias in consensus with a third reviewer (VHG) to resolve possible disagreements.

## Results

### Search, screening and selection of results

The search of different databases identified 728 articles. In addition, 2 articles identified from the bibliographic citations of the selected articles. After the removal of duplicates, the titles and abstracts of 723 articles were analyzed to determine whether they met the inclusion criteria. After this second screening, which resulted in 696 articles being discarded because they dealt with subjects different from the focus of the study, 27 texts remained. Of these, 4 additional articles were excluded as reviews, and an additional 2 were excluded as case reports. Finally, 21 articles were included in the systematic review. The search, screening and selection process is reflected in the PRISMA flow chart (Fig. 1).

### Description of included studies

In Additional file 1: Table S1 and Table 3, the characteristics of the 21 articles included in this systematic review are presented. Nineteen articles analyzed chronic effects of the WB-EMS, and 2 analyzed acute effects. Of the studies that analyzed chronic effects, 6 are part of a sequence of experimental phases that are called Test I [25], Test II [26]and Test III [2, 16, 17]. Test I [25] is described in a study. Test II [26] is documented by a study in German but is also detailed in a review [27] that the authors perform after these first two phases. Finally, three studies comprise Test III [2, 16, 17]. On five occasions, two or more articles refer to the same experimental phase: [28] with [29]; [17] with [16] and with [2]; [30] with [31]; [18] with [32]; [33] with [34, 35] with [36]. The remainder of the studies refer to independent experimental phases.

### Characteristics of the sample

In the studies that are part of this review, a total of 505 subjects were analyzed, including 310 men and 195 women. A total of 178 were subjects with a certain level of training, whereas 327 were sedentary. For the most part, the studies that analyzed chronic effects are comprise samples from postmenopausal women. In Test I [25] (*n* = 30), the participants were trained women. In Test III [2, 16, 17] (*n* = 60) and [33–36] (*n* = 100), the participants were sedentary individuals with sarcopenia or/and osteopenia. In other studies [30, 31] (*n* = 75), the sample population suffered from sarcopenic obesity and metabolic syndrome. In Test II [26] (*n* = 28), the sample population included sedentary men with metabolic syndrome. In another study [37] (*n* = 41), the sample population included sedentary men but with a good health. In six other studies [38] (*n* = 9), [39] (*n* = 18), [40] (*n* = 26), [41] (*n* = 19) and [18, 32] (*n* = 20), the subjects were trained men. In [42] (*n* = 30 woman and *n* = 34 men), the participants where sedentary young people (20–25 years). Finally, in the experimental phase of Filipovic [28, 29] (*n* = 15), participants were professional soccer players. Due to the existence of articles from the same experimental phase, in this review, the subjects of each of these studies have been counted only once to avoid incurring a risk of bias.

### Interventions

In the Test I [25], all the participants underwent two supervised sessions of 60 min weekly and another two sessions of 25 min at home (these sessions consisted of aerobic exercises, multilateral jumps and strength exercises 1–3 sets, 6–12 repetitions, 70–85% 1RM). In addition, the electrical stimulation group underwent a weekly training session with 15 exercises to strengthen the larger muscle groups. In Test II [26], the experimental group performed 15 min of elliptical exercise at 70–85% of the maximum aerobic speed in addition to 15 min strength exercises for the main muscle groups with a short range of movement. All of these exercises were performed with superimposed WB-EMS. The control group (CG) stretched on vibratory platforms in 18-min sessions with a frequency of 30 Hz, an amplitude of 1.7 mm and an acceleration of 1.3 to 2.2 g. In Test III [2, 16, 17], both the experimental and the control group underwent 10–14 dynamic exercises without additional load in each session (1–2 sets of 8 repetitions). The experimental group trained uninterruptedly during the study in three sessions every two weeks, whereas the CG trained a 60-min session weekly for 2 periods of 10 weeks separated by a 10-week period of inactivity. In the study by Kemmler et al. [37], high-intensity training (HIT) was compared with another training regimen with WB-EMS. The HIT consisted of sessions of 10/13 exercises between strength machines and core exercises. In the first two weeks, 2 sets of 15 repetitions were performed. In the following two weeks, two sets of 8–10 repetitions and in the remaining four weeks, muscle failure was addressed by further decreasing the number of repetitions per set from 8 to 3. The experimental group underwent 1–2 sets of 6–8 repetitions of 12 core-strengthening exercises with WB-EMS superimposed in standing position without an additional load.

Another experimental phase [30, 31] included a CG that did not undergo any type of training. The experimental group performed slight movements of the upper and lower limbs while in a half-lying supine position without additional load but with a superimposed WB-EMS. In this study, a second experimental group was included for which supplementation was provided. In Filipovic et al. [28, 29], the entire sample performed 3 sets of 10 repetitions of squats, but the experimental group did so with superimposed WB-EMS. In Wirtz et al. [18, 32], the entire sample performed 4 sets of 10 repetitions: the first at 50% of 10RM and the other three at 100% of 10RM. The only difference in their treatment was the application of the WB-EMS superimposed on the experimental group. In the study by Wolfgang Kemmler et al. [41] about acute effects on caloric expenditure, all the study subjects performed the same protocol that was already applied in Test I [25] with the exception that the experimental group performed it with WB-EMS superimposed without any additional burden. In Kemmler et al. [33–36], the experimental group performed the same exercises described in Test II with superimposed WB-EMS in addition to receiving a protein supplement. A second experimental group only received the protein supplementation. The control group did not perform any type of exercise and did not receive protein supplement. In Jee [42], the experimental group performed ten types of isometric exercises with WB-EMS superimposed, whereas the CG performed the same exercises without WB-EMS. In De la Cámara et al. [38], all participants performed the same training in three separate days under identical conditions, but different recovery methodologies were applied each day. One of these methodologies was the application of WB-EMS in the prone supine position.

### Current parameters and intensity

In most studies, the frequency of the current was 85 Hz. In Test I [25], after 10 min with this frequency, 7 Hz was applied for an additional 10 min. In Test II [26], the applied frequency was the inverse as it involved 15 min at 7 Hz followed by 15 min at 85 Hz. In Kemmler et al. [33–36], the applied current was 85 Hz during the entire session. Amaro [39] applied the current following an undulating periodization model in which the frequency varied from 12 to 90 Hz. The chronaxie or pulse width was of 350 μs in all cases. The parameter that varied the most during the studies was the duty cycle, which indicates the relationship between contraction time and resting time. Although most studies involve 4–6 s of work every 4 s of rest, Filipovic et al. [28, 29] proposed 4 s of work every 10 s of rest. In contrast, Wirtz et al., [18, 32] proposed 5 s of work every 1 s of rest. The rise ramp is the time that elapses from the beginning of the electrical stimulus to its maximum intensity, where i was 0 s n all cases. The same occurred with the descent ramp. To understand the internal load that caused the current in the subjects, most of the studies used the Borg scale with the exception of Test I [25] and Test II [26]. In these tests, a scale 1 to 7 was used with 1 representing the lowest current intensity perception and 7 the highest. Wirtz et al. [18, 32] performed a test to understand the pain threshold to apply an intensity corresponding to 70% of said threshold of pain during the intervention. However, Wolfgang Kemmler et al. [37] applied the current to an intensity equivalent to “hard = 15” or “very hard = 17” on the Borg scale in which the maximum level is 20. In Jee [42], as the exercises were isometric, they were able to apply a current intensity corresponding to the maximum tolerance. All studies used the same electrical stimulator device (MIHA bodytec® (Augsburg, Germany) except for Jee [42], which used Miracle® suit (Seoul, Korea). Both devices generate a type of bipolar, rectangular and biphasic current.

### Risk of bias

**Fig. 2:**
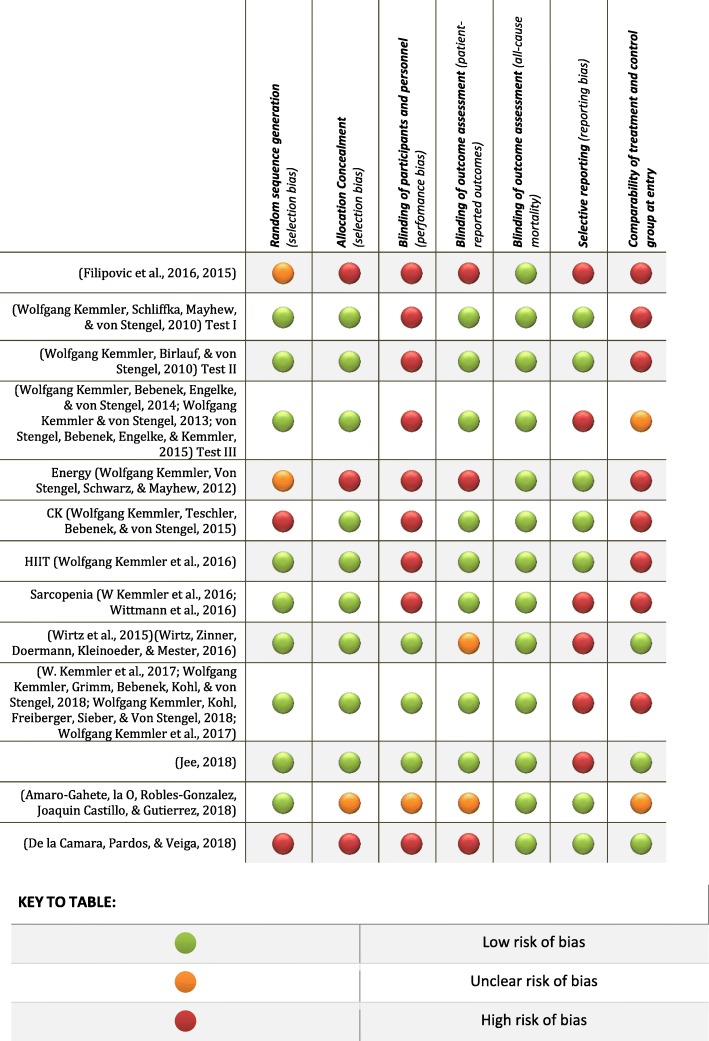
Risk of bias summary by item and study. Green marker: Low risk of bias; Orange marker: Unclear risk of bias; Red marker: High risk of bias

**Fig. 3:**
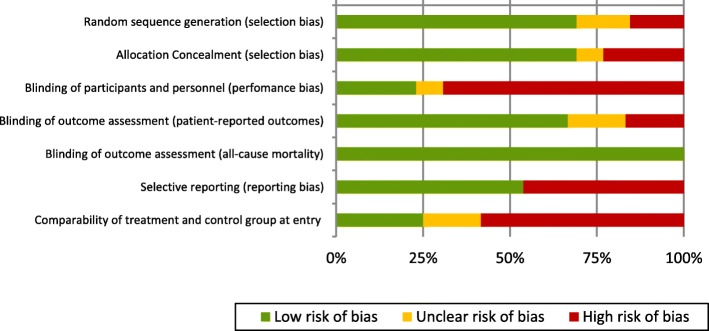
Risk of bias graph by item. Green marker: Low risk of bias; Orange marker: Unclear risk of bias; Red marker: High risk of bias

Figure 2 analyzes the different items used in the analysis of the risk of bias in each study. In Fig. 3, each type of risk of bias is studied at a general level.

#### Random sequence generation (selection bias)

All of the studies perform a randomization of the sample; however, in some cases, the methodology could incur some methodological error. In the case of Filipovic et al. [28, 29], there is a possible risk of selection. The author mentions that despite performing sample randomization, it allows a subject of the study to choose their membership in the CG given the discomfort that the WB-EMS imposes on them. In De la Cámara et al. [38] and Kemmler et al. [41], experimental and control groups include the same subjects who perform the intervention under different conditions, so the study is not truly randomized.

#### Allocation concealment (selection bias)

In Wolfgang Kemmler, et al. [40], the sample was decompensated due to the enormous numerical difference between the subjects that comprised the CG and the group of WB-EMS.

#### Blinding of participants and personnel (performance bias)

In the study by Filipovic et al. [28, 29], it is understood that if the subjects could choose their membership in the CG, it would be very likely that the entire sample knew the protocol of the study and the group to which they belonged. In such a case, there would not be a blinding of the participants with a possible placebo effect.

#### Blinding of outcome assessment (patient-reported outcomes)

In the studies by Filipovic et al. [28, 29] and Amaro et al. [39], there is no evidence that there was a blinding of the evaluators, so a risk of bias exists. On the other hand, in three additional studies [38, 40, 41], the crossover design was used, so the complete sample was at the same time. The sample of the control group, after a wash-out period, was the same in experimental group, so blinding of the evaluators was not possible.

#### Selective reporting (reporting bias)

The results are presented partially in different articles in six of the experimental phases that are analyzed in this systematic review: Filipovic et al. [28, 29], Test III [2, 16, 17], (W Kemmler et al. [30, 31], Wirtz et al. [18, 32], Kemmler [33–36] and Amaro et al. [39]. This method could incur a possible risk of notification bias given the possibility that it is mistakenly understood that these are different experimental phases, which would magnify the results of the same study.

In Jee [42], intragroup analysis is performed for psychophysiological variables but not for cardiopulmonary variables, which prevents the analyses of the effectiveness of WB-EMS in such variables.

#### Comparability of treatment and control group at entry

This type of risk of bias is more conflicted with the rigorous scientific procedure. In Test I [25], WB-EMS is applied in an extra weekly session to the experimental group in which the participants performed a series of exercises that were not practiced by the subjects of the CG, so it is impossible to objectively determine the isolated effect of WB-EMS. In the Test II [26], the WB-EMS is not the only differentiating variable in both groups because the CG performs stretching work on a vibration platform instead of performing the same exercises as the experimental group. Thus, it is not possible to determine the isolated effect of WB-EMS. The same limitations are noted in Kemmler [30]. In this study, the CG did not perform any exercise, whereas the experimental group performed upper and lower limb movements while electrical stimulation occurred. In Test III [2, 16, 17], it seems that the training of both groups is based on the same exercises. However, the WB-EMS group performed three sessions every two weeks, whereas the CG group completed a weekly session of 60 min per week and rested 10 weeks during the course of the study. Thus, the treatment is not equal in terms of volume and distribution of the loads in both groups. In the study by Wolfgang Kemmler [40], the group treatments were not the same because the objective was to compare the effect of two different activities. Thus, one group ran a marathon, and the other group underwent WB-EMS training. A similar experimental setup was noted in Amaro et al. [39]. In this study, the two experimental groups performed strength exercises during their weekly session of WB-EMS, but the control group exclusively performed aerobic running throughout the study. In the study by Kemmler [37], a group performed exercises in the context of WB-EMS that differed from those used by the CG that relied on high intensity training (HIT) with guided motion strength machines. This difference could perhaps allow comparisons of the effects of the two trainings but could not determine the adaptations that WB-EMS causes alone. Finally, in the study by Kemmler [33–36], the CG did not perform the same exercises as the experimental group (in fact, CG did not perform any type of exercise). Thus, it is not possible to determine whether the possible improvements are attributed to the exercises or to WB-EMS; thus, the effect of WB-EMS alone cannot be analyzed.

### Outcome measures

#### Anthropometric parameters

Research on WB-EMS has identified minimal effects in relation to anthropometric parameters. In Filipovic et al. [29], Kemmler et al. [37], and Wirtz et al. [32], no significant changes were found. In Test I [25], body weight decreases (− 1.9 ± 1.7 kg, *p* = 0.001). However, body weight is also decreased in the CG (− 0.9 ± 1.5 kg, *p* = 0.025), and no significant differences are noted between both groups. No changes were noted in Test II [26] and Test III [2, 16, 17]. Total body fat is reduced in Kemmler et al. [33–36] (− 2.05 kg (− 1.40 to − 2.68), p = 0.001), but the difference between WB-EMS&P and the protein groups was borderline nonsignificant (*p* = 0.051). Regarding the sum of skinfolds, in Test I [25], a decrease is observed (− 8.6%, *p* = 0.001). However, the value increases (1.4%) albeit nonsignificantly in the CG. The waist circumference is reduced in this same study (− 2.3%, p = 0.001), whereas an increase is noted in the CG (1%, *p* = 0.106). The hip circumference decreases in Test I (− 2.3%, p = 0.001) and in the CG (1.3%, *p* = 0.008). The waist circumference decreases (− 5.7 ± 1.8 cm, p = 0.001) in Test II [26] and in the CG (− 3.0 ± 2.0 cm, *p* = 0.006). In Test III [2, 16, 17], the waist circumference decreases (− 1.1 ± 2.1 cm). However, a large deviation is observed, which is also noted in the increase observed in the CG (1.0 ± 2.8 cm). The level of significance of these data is not provided. Similar findings are noted in Kemmler et al. [33–36] where waist circumference decreases (− 1.94 cm (− 1.44 to − 2.44), *p* = 0.001) with a significant group difference (*p* = 0.001) between the treatment group and the CG (− 0.10 cm (.46 to −.67)). A high deviation also occurs in the study by Kemmler et al. [31], where waist circumference is reduced in the WB-EMS group (− 1.5 ± 2.3%, *p* = 0.004) and the CG (− 0.02 ± 2.26%, *p* = 0.963). Muscle mass increases in Test I [25], Test II [26], Test III [2, 16, 17], Kemmler et al. [31] and Kemmler et al. [33–36]. However, in all cases, the effect is minimal with large deviations and a low level of significance. Similar results were noted for appendicular muscle mass in Test I [25], Test II [26] and Test III [2, 16, 17]. Regarding fat mass, Additional file 2: Table S2 demonstrates that the changes are almost imperceptible, and large deviations are noted in Test II [26], Test III [2, 16, 17] and Kemmler et al. [31]. In Test III [2, 16, 17], the evolution of bone mineral mass is measured but no effects were observed.

#### Strength parameters

Filipovic et al. [28, 29] was the only study that measured the 1RM, observing an increase of 22.42 ± 12.79% (*p* < 0.01) after fourteen weeks of WB-EMS without changes in the CG. According to the authors, this gain in strength explains the improvement in sports skills, such as the linear 5-m sprint (− 0.3 s, *p* = 0.039), 10-m sprint with changes of direction (− 0.18 s, *p* = 0.024), one-step chute speed (+ 9.9 km/h, *p* = 0.001), and squat jump (+ 2.9 cm, *p* = 0.021). Most of the measurements that are made to study the evolution of strength analyze its manifestation in the isometric muscle contraction regime. In Test I [25], the isometric maximal strength improved (9.9%; *p* = 0.015) in the extensors of the leg and extensors of the trunk (9.6%; *p* = 0.001), which is parameter that was reduced in the CG (− 6.4%, *p* = 0.054 and-4.5%, *p* = 0.106). In Test II [26], improvements in power (+ 10 ± 7%, p = 0.01) and isometric maximal strength (+ 15 ± 11%; p = 0.01) of the leg extensors were observed, whereas both parameters decreased (+ 3 ± 4% and − 0.5 ± 6%) in a nonsignificant manner in the CG (*p*-values not provided). Increases were noted in Test III [2, 16, 17] (9.1 ± 11.2%, *p* = 0.002) and the CG (1.0 ± 8.1%, *p* = 0.631). However, large standard deviation was noted and the data lacked significance. Similar results were noted in Kemmler et al. [37] as presented in Additional file 2 Table S5. Handgrip strength increased in the study by Kemmler et al. [33–36] (1.9 kg (0.99 to 2.82), *p* = 0.001)) with a small size effect and large deviations, and non-significant differences were observed between the treatment group and the CG. In the same study, maximum dynamic strength “leg-press” increases (189 ± 129 N, *p* = 0.001), but the difference between WB-EMS&P and the protein group was not significant.

#### Energy expenditure and cardiovascular system

Kemmler [41] conducted a study of caloric expenditure by indirect calorimetry of a 16-min session of low intensity strength exercises performed by young subjects (26.4 ± 4.3 years), revealing an increase of 17% with superimposed WB-EMS (412 ± 60 kcal · h-1 versus 352 ± 70 kcal · h-1, *p* < 0.01). However, in Test I [25], no significant increase in resting metabolic rate was observed after 14 weeks of training.

#### Blood parameters

Filipovic et al. [28, 29] did not report significant differences in the evolution of blood parameters, such as the concentration of red blood cells, platelets, white blood cells or hemoglobin. The authors of this experimental phase report that in week 7 and 14 of their intervention, an increase (*p* < 0.05) in the size and deformability of red blood cells was observed. These results indicate an increased capacity for the transport of oxygen to muscular cells. However, the effect size is not recorded. In Kemmler et al. [31], no changes in triglycerides, glucose and cholesterol were observed after 26 weeks of training with WB-EMS. Similar results were noted in Wirtz et al. [18] given that no differences were noted in the analysis of the evolution of testosterone, cortisol and growth hormone. A positive aspect of this study is the absence of pre-post changes in other parameters that could indicate overtraining in cases with observed high values, such as lactic acid and creatine kinase (CK) activity. Kemmler et al. [33–36] observed significant changes in the total cholesterol/HDL-C ratio (− 0.31 index (−.15 to −.47), *p* = 0.001) and in the protein group who did not train with superimposed WB-EMS.

#### Psychophysiological parameters

Jee [42] is observed a positive effect of WB-EMS in psychophysiological variables using a scale from 1 to 10. Soreness (− 4.16 ± 1.20), anxiety (− 3.75 ± 0.91), fatigability (− 3.33 ± 1.01) and sleeplessness (− 4.88 ± 1.13) were significantly changed (p = 0.001). Control group data were not provided.

## Discussion

The aim of this systematic review was to determine the effects of WB-EMS. Taking into account the enormous interest in this training methodology in recent years, a review that complies and objectively verifies the knowledge obtained on the subject to date is needed to clarify the state of the matter.

Studies in the field of WB-EMS are beginning to increase. In addition, due to the enormous interest in the use of this training tool that can become very dangerous if used incorrectly, a guide for its correct use has been created in a very appropriate and timely manner by mainly appealing to common sense [43]. However, to our knowledge, the research body is scarce, and the existing studies lack the amount of evidence necessary to draw solid conclusions about the effectiveness of training with WB-EMS and adequate technical guidelines for its use and management in different contexts and needs.

Many of the studies published to date have been performed with population groups with very determinant diseases, indicating that these studies lack a high level of external validity. Taking this limitation into account, in their systematic review on the effects of electrical myostimulation, Filipovic et al. [9] report a high correlation between the intensity of the current and the effects of EMS. It is believed that this type of population with special needs and a delicate state of health may not be the most suitable for electrical stimulation training. Similarly, it is not believed that this type of sample is the most adequate to reproduce the physically demanding current parameters that have been typically applied given the influence of certain brands of electrical stimulators that commonly provide a frequency of 85 hz. In his review, Filipovic [4] concludes that a current of 50 hz is sufficient for the activation of type II fibers and strength work. In fact, previous studies indicate the need to minimize the frequency of the current as much as possible given that its increase is accompanied by an increase in muscle fatigue [44]. Therefore, it seems that the electrical stimulus chosen in some of the studies is not the most commonly recommended for people with atrophy of their muscular system and a sedentary lifestyle.

On the other hand, what has been demonstrated in studies with local EMS is the effectiveness of training with EMS as a means for functional improvement in elderly populations [45, 46], which makes the appearance of new research on WB-EMS in these population groups necessary with parameters more adapted to their needs.

Regarding the time-to-rest electric stimulus ratio and considering what was said above, it seems that in studies of WB-EMS where populations exhibited some type of physical handicap, these populations were exposed to duty cycles of excessive density, i.e., close to 50%. In contrast, 20–25% is recommended to guarantee sufficient recovery and strength adaptation [9]. In the study by Wirtz et al. [18] of soccer players, a strength work and a duty cycle of 83.3% was proposed. The demanding density of these protocols, which do not guarantee a necessary rest, could perhaps be the cause of a poor evolution of the strength or the absence of improvement as an adaptation to WB-EMS training in this study.

The application of the WB-EMS is typically performed in sports centers or beauty centers where training sessions last for 20 min. This is a controversial issue. Filipóvic et al. [9] consider that 20 min is highly advisable and a sufficient time period to increase the levels of strength and the physical skills that are derived from it, whereas other study conclude that a classic 20-min training session does not seem the most appropriate for improvement of sports skills or the rehabilitation of injuries [47]. It must be taken into account that depending on the parameters of the current, the muscular fatigue that is generated can vary enormously [11]. Thus, establishing such a short fixed time without remission would determine the characteristics of the training session. That a training session with EMS or WB-EMS should only contain muscle contractions combined with the current could be a common mistake. In this sense, to conceive EMS and WB-EMS as a resource among the many others available to the professional instead of converting the currents into the objective of the session would be appropriate and enriching.

Regarding the anthropometric results obtained in the studies analyzed, no statistically conclusive evolutions have been observed to date. In addition to not recording feeding control in any of the cases, the results reveal a small effect size with a large standard deviation, and the values are significant in a limited number of cases. However, it is possible that in the future, research on WB-EMS will provide more positive results in this field. It should be considered that EMS applied simultaneously to aerobic exercise can contribute to the reduction of fat tissue to a greater extent than aerobic exercise alone [48]. However, it should be noted that this study made its assessments through the analysis of skinfolds, suggesting that research with more precise assessment techniques for the evaluation of anthropometric parameters and their evolution before training with EMS and WB-EMS is needed.

In the context of different exercises with the same level of maximum oxygen consumption (VO2), EMS causes a significant increase in lactic acid and glucose consumption, suggesting that the current increases energy consumption and the oxidation of carbohydrates to a greater degree compared with that produced by voluntary contraction [49]. In the study by Kemmler et al. [41], a 17% increase in energy consumption was observed during exercise performed with simultaneous WB-EMS. This minimum difference could not justify its use for this purpose although the WB-EMS involves a greater area of electrical stimulation than local EMS. The authors note that they potentially underestimated the effect of WB-EMS given that their measurement of energy consumption through VO2 is only valid in steady state situations. However, in this study, they do not indicate at any time that the participants received a familiarization session with WB-EMS prior to the experimental phase. A previous study demonstrated the need for at least one EMS session prior to the study to minimize the muscle damage produced by the current and favor the familiarization of the subjects to the electrical stimulus [50]. It is possible that this limitation could have led to the fact that the intensity of the current with which the participants performed the exercise was substantially lower than they could sustain without risk if the participants had completed a phase of previous adaptation. Therefore, the potential effects of WB-EMS on energy consumption could have been minimized in this experimental phase.

With regard to the effects on strength, only two studies analyzed the effect of the WB-EMS in the dynamic 1RM. Kemmler et al. [33–36] found a significant increase (9.5%, *p* = 0.001). In the study by Filipovic et al. [28, 29] of trained subjects, they confirmed an increase (22.42% ± 12, 79). This increase is similar to that observed by Willoughby & Simpson [51] (26.3%) in a study that also simulated the application of currents with dynamic voluntary contractions three days a week but with local EMS. However, in other studies with similar methodology for local EMS, lower increases in the dynamic strength of the lower limbs were observed after training for three days a week for 12 weeks (+ 15.0 +/− 8.0%, *p* < 0.001) [52]. Others have found that the 1RM increased 40.2% due to local EMS with four workouts per week for four weeks [53]. The remaining studies assessed in this review analyzed the isometric maximal strength without finding effects with a substantial effect size. In many cases, the standard deviation is greater than the effect size, and significant results are extracted in rare cases. Considering that a significant increase of 22% in the (IS) has been reported after a training period combining isometric and dynamic contractions with local EMS [54], it is expected that with evolution and development of the technology, application protocols of WB-EMS will offer more positive results in the future.

Regarding the effects on blood parameters, none of the studies analyzed in this review reported significant changes after training with WB-EMS with the exception of total cholesterol/HDL-C and creatine kinase activity. Regarding the total cholesterol/HDL-C ratio, Kemmler et al. [33–36] observed a decrease of (− 0.31 (−.15 to −.47), *p* = 0.001), but the protein group experienced an even greater decrease (− 0.34 (−.21 to −.47),p = 0.001). Thus, this effect could not be attributed to WB-EMS. With regard to creatine kinase activity in blood, an increasing number of case reports describing situations of rhabdomyolysis with an alarming increase in creatine kinase immediately after exercise with WB-EMS has been reported [20–22]. As Stöllberger C. and Finsterer J. [43] indicated in their review of the side effects of the WB-EMS, rhabdomyolysis occurred after a first WB-EMS training session in most cases. In addition, the current parameters had been physically demanding, especially regarding intensity [22] and application time [21]. These findings indicate that the principle of load progression training was not respected with the completion of a phase of previous adaptation to the current to minimize muscle damage. It has been observed that after four sessions of WB-EMS, creatine kinase activity decreases significantly as a consequence of the adaptation of the muscular system to WB-EMS [40]. This finding implicitly implies that exercise with WB-EMS should be always performed under the supervision and direction of a technician trained and updated in the advances of this technology to avoid unnecessary risks caused by ignorance and mere lucrative desire.

Following the analysis of existing literature on the issue, it is deduced that the emergence of new studies with rigorous and consistent methodologies and protocols is necessary to shed light on WB-EMS and to objectively prove its effectiveness. In addition, adequate protocols should be established to individualize training with currents and make it a safe practice.

## Conclusions

The findings of this review suggest that more studies are needed that include populations without special needs to establish the effects produced by the different current parameters in WB-EMS. A limited number of studies on WB-EMS are available. Many of the existing investigations have been performed with population groups with special needs and, therefore, lack external validity. Many of the existing studies lack the amount of scientific evidence necessary to draw reliable conclusions about the effects of WB-EMS. More studies are needed in populations without special needs to establish the effects produced by the different current parameters in WB-EMS. It would be appropriate for the relevant legislators to regulate the application of WB-EMS to ensure its consistent use under the direction of qualified and authorized professionals, including sanctioning the negligent use and free assumption of risks of noncertified services.

### Study limitations

- Nonrandomized studies have been included.

- There are few studies and, thus, a high risk of bias.

## Additional files

Additional file 1: **Table S1.** Characteristics of the studies included in the review. (XLSX 15 kb)

Additional file 2: **Table S2.** Results of the analyzed studies. (XLSX 27 kb)

## Abbreviations

BMD: Bone mineral density
CG: Control Group
CK: Creatine kinase
CMJ: counter movement jump
EMS: Electrical Muscle Stimulation
EXP & P: Experimental Group with Protein Supplementation
EXP: Experimental Group
GH: Growth hormone
HIT: High-intensity Training
IMS: Isometric Maximal Strength
MRI: Maximum Repetition
R: Recovery Between Series
RBD: Red blood cell deformability
RM: Maximal Repetition
RPE: Rated Perceived Exertion
SJ: Squat Jump
VO2 max: Maximum Oxygen consumption
VO2: Oxygen consumption
WB-EMS: Whole-Body Electromyostimulation
